# Iron-deficiency Anemia in Children with Febrile Seizure: A Case-Control Study

**Published:** 2014

**Authors:** Fateme GHASEMI, Fateme VALIZADEH, Nadere TAEE

**Affiliations:** 1 Nursing Faculty, Lorestan University of Medical Sciences, khorramabad, Iran; 2 PhD student of Nursing Education, Ahvaz Jundishapur University of Medical Sciences, Ahvaz, Iran; 3 Pediatric Department, Lorestan University of Medical Sciences, khorramabad, Iran

**Keywords:** Iron-deficiency anemia, Febrile seizure, Febrile disease

## Abstract

**Objective:**

Considering the recurrence of febrile seizure and costs for families, many studies have attempted to identify its risk factors. Some recent studies have reported that anemia is more common in children with febrile convulsion, whereas others have reported that iron deficiency raises the seizure threshold. This study was done to compare iron-deficiency anemia in children with first FS with children having febrile illness alone and with healthy children.

**Materials & Methods:**

This case-control study evaluated 300 children in three groups (first FS, febrile without convulsion, and healthy) in Khoramabad Madani Hospital from September 2009 to September 2010. Body temperature on admission was measured using the tympanic method. CBC diff, MCV, MCH, MCHC, serum iron, plasma ferritin and TIBC tests were performed for all participants. Data were analyzed by frequency, mean, standard deviation, ANOVA, and chi-square statistical tests. Odds ratios were estimated by logistic regression at a confidence level of 95%.

**Results:**

Forty percent of the cases with FS had iron-deficiency anemia, compared to 26% of children with febrile illness without seizure and 12% of healthy children. The Odds ratio for iron-deficiency anemia in the patients with FS was 1.89 (95% CI, 1.04-5.17) compared to the febrile children without convulsion and 2.21 (95% CI, 1.54-3.46) compared to the healthy group.

**Conclusion:**

Children with FS are more likely to be iron-deficient than those with febrile illness alone and healthy children. Thus, iron-deficiency anemia could be a risk factor for FS.

## Introduction

Febrile seizure (FS) is the most common convulsive disorder in children, which affects 2-5% of children aged 3 to 60 months (1). Although FS is benign and rarely leads to brain damage, it causes emotional, physical, and mental damages, which are stressful for parents, and affects families’ quality of life (2, 3). Many studies have tried to find its risk factors, because of its relation to epilepsy in 2-4% in future, the fact that it can lead to hospitalization, costs for families and the society, and likelihood of recurrence (30% and 50% after the first and the second occurrences, respectively) (2, 4).

Some recent studies have reported that iron-deficiency anemia (IDA) could be a risk factor for FS, because the latter is more common in children under two years of age and IDA is also common in children of the same age. 

Due to the presence of iron in hemoglobin structure, it plays a crucial role in the transport of oxygen to different tissues such as the brain (5). Iron deficiency (ID) reduces the metabolism of some neurotransmitters, such as monoamine and aldehyde oxidase (6, 7). Several lines of evidence led to the hypothesis that iron may have a role in the onset of a convulsion. However, the studies carried out so far have reported conflicting results. Some studies have reported that in the patients with ID, febrile convulsion is significantly higher than that in control group (5,8-11). On the contrary, some authors have concluded that the risk of FS in anemic children seems to be less than that in children without FS (12) and that ID can be a protective mechanism against convulsions by increasing the convulsion threshold, and thus iron supplements should be given with caution to the children (13). Other studies have shown that ID plays no role in pediatric FS (14, 15). 

Since the relationship between IDA and FS is not yet determined, chance or other unknown factors can be considered as causes (16). Considering the above results and since no study has been conducted in Lorestan Province on the mentioned relationship, the present study was carried out to compare the IDA rates in FS children with those in the febrile children without seizure and healthy children.

## Materials & Methods

This is a descriptive-analytical case-control study, which was conducted on 300 participants in three 100-person groups (FS, febrile without seizure, and healthy) in Shahid Madani Hospital in Khorramabad city during September 2009 to September 2010. The case group consisted of children, aged 5 months to 5 years, with normal development, who were taken to the hospital for the first FS (FFS) and were selected steadily and gradually during the study period. Samples with electrolyte imbalance (sodium, calcium, etc.), hypoglycemia, meningitis, encephalitis, neurological deficiencies, and history of seizures (with or without fever) were excluded from the study. For each child with febrile convulsion, two children were selected in the control groups: one with a similar febrile illness, who was admitted in the hospital at about the same time as the subject, but did not show seizure, and another was a healthy child who was referred to a health care facility for medical services. Children of the control groups were selected using an objective-based method and matched with the cases in terms of age (with a maximum difference of one month), gender, and place of residence (urban or rural). In addition, the members of the febrile group were matched to those of the case group in terms of the background disease causing fever. In all cases, after the goals and the voluntary nature of the study were explained to the parents, they were asked to sign a consent form. The study was also confirmed by the Ethics Committee of Lorestan University of Medical Sciences. 

Children’s personal information, including age, gender, place of residence, family history of seizure, and history of taking iron supplements, were collected from parents through an interview questionnaire. The type of FS for case group was extracted from their medical records. In both case group and the group of febrile children without convulsion, body temperature was also measured and recorded using a tympanic thermometer (Micro life IR 100, Germany). After making sure that the participants had not taken iron supplement in the last three days, a 5 ml fasting blood sample was taken and sent to the laboratory. The blood tests of CBC diff, MCHC, MCH, MCV, iron and serum ferritin levels, and TIBC were done in one using a 18-parameter Sysmex KX-21N hematology analyzer, a gamma counter Genesis-Camna1 by radioimmunoassay (RIA) kit (for measuring ferritin), and a 717 Hitachi auto-analyzer set (for measuring serum iron and TIBC through chromatography). All the blood tests were performed by a laboratory technician who was not informed of the purpose of the study. 

The diagnosis of anemia was made by a pediatrician who studied the results of tests without knowing the groups to which each of the test results belonged. For children aged 6 months to two years, IDA was defined as Hb<10.5g/dl, hematocrit (Hct)<33%, MCV<70 fL, MCH<23 pg, MCHC<30 g/dL, and RBC<3.7×10^6^ cell/ mm3. For 2-5-year-old children, IDA was defined as Hb<11.5 g/dL, Hct<34%, MCV<75 fL, MCH<24 pg, MCHC<31 g/dL and RBC<3.9×10^6^ cell/mm³. MCHC, MCH, MCV, and peripheral blood smear were used to rule out other causes of anemia. 

The normal level of serum iron was determined as Fe>40 μg/dL for children younger than one year and Fe>50 for children over one year of age. The normal range of ferritin was established as more than 12 ng/dL for healthy children and more than 30 ng/dL for children with infection. The normal range of TIBC was considered 210-430 μg/dL. The normal transferrin saturation percentage was considered higher than 15% (4,17).

The collected data were analyzed using descriptive statistics, including frequency, percentage, mean and standard deviation, as well as analytical statistics, such as chi-square (for comparison of qualitative variables) and the analysis of variance test and t-test (for comparison of quantitative variables). In addition, odds ratios were calculated by logistic regression at a confidence level of 95%.

## Results

In the case of the personal characteristics, no significant differences were found among the groups in terms of age, gender, place of residence, and the type of nutrition during infancy. However, there were significant differences between the study groups regarding the family history of convulsion and history of taking iron supplements. 

Furthermore, the febrile convulsion group and the group of fever without convulsion had no significant difference in terms of the disease causing fever, but body temperatures were significantly different at the time of admission (Table 1). In the febrile convulsion group, the ratio of boys to girls was almost 2 to 1. The type of convulsion was simple in 87% of the cases and complex in the remaining 13%. In the febrile convulsion group, most of the members (54%) belonged to the age group of less than two years (17% were less than one year, 37% between 1 and 2 years, 22% between two and three, and 24% over three years). The diseases causing fever in both children with and without seizure were respiratory and digestive infections. The means of Hb, Hct, RBC, serum iron and ferritin, TIBC, and transferrin saturation were significantly different among the groups. However, no significant differences were found among the groups in terms of MCV, MCH, and MCHC (Table 2). The presence of IDA was 40% in the convulsion group, 26% in the group with fever without convulsion, and 12% in the healthy group. The chi-square test indicated a significant difference among the groups, and 51.3% of the subjects with IDA had experienced seizures (Table 3). A comparison of the odds ratio of IDA for different groups is shown in Table 4.

**Table1:** Personal Information about the Study Children in the Three Groups of Febrile Convulsion, Fever without Convulsion and Healthy

**Group**		**Febrile seizure n(%)**	**Febrile without seizure n(%)**	**Healthy n(%)**	**p-value**
**Gender**	**Boy**	66(66)	59(59)	58(58)	0.45^a^
	**Girl**	34(34)	41(41)	42(42)	
**Residence Place**	**Urban**	73(73)	67(67)	69(69.9)	0.65 a
	**Rural**	27(27)	33(33)	30(30.1)	
**Family history of seizure**	**Yes**	22(22)	10(10.1)	1(1)	0.000 a
	**No**	78(78)	89(89.9)	97(99)	
**History of taking iron supplements**	**Yes**	74(76.3)	68(68.7)	86(88.7)	0.003 a
	**No**	23(23.7)	31(31.3)	11(11.3)	
**Infancy nutrition**	**Breast milk**	82(82)	82(82)	74(74.7)	0.54^ a^
	**Formula**	4(4)	8(8)	9(9.1)	
	**Both**	14(14)	10(10)	16(16.2)	
**Infectious disease type**	**Respiratory**	60(60)	57(57)	-	0.91 a
	**Gastroenteritis**	39(39)	42(42)	-	
	**Others**	1(1)	1(1)	-	
**Age (month)**		27.13±15.74 d	26.18±17.04	27.58±14.33	0.77^b^
**Temperature**		38.89±0.59 d	38.50±0.48		0.000^c^

a . Chi Squar;

b .Analysis variance;

c . T-Test;

d . Mean + standard deviation

**Table 2:** Mean And Standard Deviation of Different Indices of Iron Deficiency Anemia the Study Children in the Three Groups of Febrile Seizure, Fever without Convulsion and Healthy

**Group**	**Healthy**	**Febrile without Seizure**	**Febrile Seizure**	**F** a	**p-vale**
HB	12.04±1.22	11.71±1.31	11.55±1.34	4.36	0.01
Hct	35.84±2.23	34.95±3.49	34.14±2.89	8.5	0
RBC	4.52± 0.39	4.35±0.45	4.24± 0.38	12.3	0
MCV	79.50±4.45	80.50± 5.62	80.73±5.15	1.66	0.19
MCH	26.73±2.19	27±2.59	27.12±2.77	0.7	0.5
MCHC	33.54±1.39	33.49±1.54	33.57±1.41	0.13	0.88
Ferretin	49.61±74.36	86.83±83.53	77.64±69.22	6.5	0.002
Serum iron	81.37±38.11	50.51± 41.85	45.92 ±26.02	28.14	0.05
TIBC	371.37± 34.33	383.82±31.64	391.44±28.12	10.36	0
Transferrin saturation percentage	22.63± 11.84	13.70 ±12.26	12.11±7.60	27.72	0

a . ANOVA

**Table 3:** Frequency Distribution of Iron Deficiency in Children of the Three Groups of Febrile Seizure, Fever without Convulsion and Healthy

**Iron deficiency** **Group**	**Positive** **n(%)**	**Negative** **n(%)**	**X** ^2^ ** Significance**
Febrile Seizure	40(51.3)	60(27)	
Febrile without Seizure	26(33.3)	74(33.3)	
Healthy	12(15.4)	88(39.6)	
Total	78(100)	222(100)	20.37 <0.001

**Table 4:** Comparison of Iron Deficiency Anemia in the Febrile Seizure Group with Fever without Convulsion and Healthy Groups

**Compared groups**	**Odds** **ratio **a	**95** **%** ** Confidence interval**	**Significance**
Febrile Seizure with Febrile without Seizure	1.89	1.04–3.46	0.04
Febrile Seizure with Healthy Children	2.21	1.54–3.18	0.001
Febrile without Seizure with Healthy Children	2.43	1.14-5.17	0.02

a . Logistic Regression

## Discussion

In this study, the Odds ratio of IDA for the febrile convulsion group was nearly two times that of the febrile group, and slightly more than two times higher than that of the healthy group, namely, the incidence of IDA in the febrile convulsion group was significantly higher than in the other two control groups. In accordance with our research, a study by Pisacane et al. reported that anemia in their case group (30%) was higher than in hospital control group (14%) and healthy group (12%) (5). Also, in a study by Vaswani et al., 68% of cases were iron-deficient compared to 30% of controls (18). 

In a study by Sadeghzadeh et al., although anemia was not common among FS patients, ID was more frequent in these patients (19). A study by Ur-Rahman and Billoo on 30 children with febrile convulsion and 30 children with other febrile diseases indicated that IDA in their case group was significantly more common than in control group (8). A Kenyan case-control study as well as the meta-analysis of 8 case-control studies that have examined the relationship between FSs or acute seizures and ID, suggested that ID may be associated with an increased risk of FSs in children (20). Fever can worsen the effects of anemia or ID on the brain and therefore cause convulsions. In addition, anemia can be associated with the severity of febrile disease, and patients with more severe symptoms may be affected by convulsions. But, febrile convulsion usually occurs at the onset of a febrile disease, before the reduction of Hb due to the infectious disease (5). In a study conducted in Thailand, the rate of Thalassemic children with febrile convulsion was reported as being 4.4 times less than the general population of children. The researchers suggested that it might be due to higher levels and the role of iron in brain metabolism, which leads to reduced occurrence of febrile convulsion in those children (21). This study, of course, could simply assess the role of increasing iron in the reduction of febrile convulsions, and cannot be an appropriate scale to measure IDA and febrile convulsion. On the other hand, low risk of febrile convulsion in thalassemic patients could be due to several other clinical conditions that they may have.

On the other hand, some studies have reported findings that are not similar to the result of present study. For example, in Hartfield et al.’s study, ID was found to be 9% and 5% in the children of two groups of febrile convulsion and febrile without convulsion, respectively, and IDA was found to be 6% and 4% in the former and latter groups, respectively (10). Again, in Kobrinsky et al.’s study, the febrile convulsion group suffered less from ID, and it was concluded that ID could have a protective effect against febrile convulsions (10); and in a study by Bidabadi, ID in the febrile convulsion group (44%) was less than in the control group (48%), but since there was no significant difference, the protective effect of ID against febrile convulsions was not confirmed (16). The major causes that have led to different results between their and our studies may include not considering the effect of age on interpretation of the tests for ID diagnosis, difference in age and number of samples, and difference in the diagnostic criteria of IDA. In the present study, all samples of the case group suffered from febrile convulsion for the first time, but in most of the mentioned studies, some samples had a history of febrile convulsion. 

In this study, the mean plasma ferritin concentration in the healthy group was lower than that in the two groups of febrile convulsion and febrile without convulsion, but there was no significant difference between the two latter groups. In studies by Daoud et al, Rehman and Billoo, and Moeman et al., the mean plasma ferritin level in the febrile convulsion group was significantly lower than the control group, which led them to the conclusion that it can demonstrate the role of IDA in the incidence of febrile seizure (8, 9, 11). It is indeed evident that ferritin is an acute phase reactive substance in nonspecific response to any febrile disease (10). This can be confirmed by the higher plasma ferritin levels in the patient groups than in the healthy group, and fever can cause the lack of difference in ferritin levels between the two patient groups. In any case, the use of plasma ferritin cannot simply be an efficient criterion for the diagnosis of ID in febrile children. 

The advantages of this research, which are not mostly observed in other studies, are as follows: having two control groups, having several laboratory criteria for the diagnosis of ID, recruiting a pediatrician, who was blind to the groups of samples to control the blood tests. 

Some limitations of this study are: the need to study the history of treatment in a daily kindergarten or nursery school and the need to study the history of taking antibiotics and antipyretics before enrollment in the group of patients. 

According to the findings of the present study, the incidence of ID in children suffering from fever and convulsion was observed to be more than that in both fever without convulsion and healthy groups. 

**In conclusion**, our findings suggest that low serum iron levels and the presence of anemia can serve as strengthening factors for the FSs in children. Therefore, ID can be added to the list of risk factors for febrile convulsions. Accordingly, children with FSs are suggested to be monitored for diagnosis and treatment of IDA. Furthermore, it is advisable to prescribe iron supplements earlier and more carefully to children who have important and well known risk factors for febrile convulsion, such as family history of febrile convulsion. It would be worthwhile to conduct a study to follow up children with ID, who are stricken by febrile convulsions after the treatment of ID, in terms of the recurrence rate of febrile convulsions. 
